# Dynamization or Dematerialization

**Published:** 1887-11

**Authors:** 


DYNAMIZATION OR DEMATERIALIZATION. By J. B. Sunderland, M.D. Boston, 1887.

The author of this pamphlet is also the editor of the *New England Medical Gazette.* This essay was first published in that journal. The author and editor were *both* so much pleased with the essay that it was reprinted in pamphlet form for distribution.

The object of the essay is to prove that high potencies are unscientific, because neither the chemist nor the microscopist can detect crude medicine in these preparations. Neither can the chemist or the microscopist tell us why one medicine differs in its action from another medicine. Neither can they detect the causes of diseases, or tell us why one morbific poison produces one disease, another a different one. In short, there are very many things of which *science* cannot tell us, which we believe in firmly and use constantly. The morbific matter of disease and the pathogenetic powers of drugs act upon those things which the Almighty intended them to act upon. High potencies *do* cure the sick, and, so doing, serve the purpose for which they were made.

